# Effects of cultivation management on the winter wheat grain yield and water utilization efficiency

**DOI:** 10.1038/s41598-019-48962-z

**Published:** 2019-09-04

**Authors:** Yonghua Wang, Huan Liu, Yuan Huang, Jinfeng Wang, Zhuangzhuang Wang, Fengxu Gu, Minghua Xin, Guozhang Kang, Wei Feng, Tiancai Guo

**Affiliations:** 1 National Engineering Research Centre for Wheat, #15 Longzihu College District, Zhengzhou, Henan 450046 PR China; 2 Synergetic Innovation Center of Henan Grain Crops, #15 Longzihu College District, Zhengzhou, Henan 450046 PR China; 3 Agronomy College of Henan Agricultural University, #15 Longzihu College District, Zhengzhou, Henan 450046 PR China; 4 Institute of Cotton Research of the CAAS, Anyang, Henan 455000 China

**Keywords:** Plant sciences, Systems biology

## Abstract

The growth of winter wheat consumes a substantial amounts of water, and precipitation in most years cannot meet the water demand for the normal growth of winter wheat. The unsuitable irrigation strategies waste a large number of water resource, and the low water use efficiency has become the main factor limiting wheat yields. This research explored the effects of different cultivation managements on water consumption characteristics, water utilization efficiency, and grain yields of winter wheat. A field experiment, in which 4 cultivation managements including traditional cultivation management (T1), optimized cultivation management compared with T1 (T2), super high-yield cultivation management (T3) and optimized cultivation management compared with T3 (T4), was conducted during 2008–2010 to measure the above parameters. The results showed that different cultivation managements had significant effects on the total water consumption amounts and water source compositions. Total water consumption amounts in T1 and T3 managements were significantly higher than that in T2 and T4 managements, possibly from irrigation water. T2 and T4 managements remarkably increased the uptake and utilization of soil storage water and precipitation amounts. T3 and T1 managements increased and decreased water consumption in upper (0–40 cm) and lower (60–100 cm) soil layers, respectively, while effectively increased the consumption of storage water in middle and lower soil layers (60–100 cm) and yield water use efficiency (WUE_Y_), precipitation water use efficiency (WUE_P_), soil water use efficiency (WUE_S_), irrigation water use efficiency (WUE_I_), and irrigation efficiency (IE) in T4 and T2 managements were higher than those in T3 and T1, respectively. Total water consumption amounts markedly raised in T1 and T3 managements, whereas their soil storage water amounts utilization declined. T2 and T4 managements reduced irrigation water amounts and optimized the water and fertilizer supplies, resulting in significant increase in WUE_S_ and WUE_I_. Collectively, our results suggest that synergetic improving the water uptake and utilization of irrigation water and soil storage water can be the primary means to increase the grain yields and WUE.

## Introduction

Huang-Huai-Hai Plain region, in which mainly occurs a monsoon climate of medium latitude with sufficient heating, lighting conditions and convenient irrigation conditions, is the region with the most suitable ecological conditions for winter wheat growth, and wheat sowing area and total wheat output in this region rank first in China^1^. In this region, 70–80% of precipitation occurs from July to September every year, however, the remaining appears other months, a stage of wheat growth and development, does not satisfy the water requirement for wheat, and water shortage has become the main factor limiting wheat yields in this region^2,3^. Therefore, irrigation water is an important guarantee for high yields of winter wheat, whereas the current irrigation systems, e.g. unsuitable irrigation periods and amounts, waste a large amount of water resource^4^, and to improve WUE of irrigation water and precipitation has become an urgent task in this region. It has been reported that soil water content is an important factor affecting the water consumption of wheat and the soil water efficiencies in different soil layers, what’s more, irrigation effectively changes the water content in the soil profiles, and affects the water uptake and utilization of wheat, whereas the excessive use and severe deficit of soil water obviously decrease WUE_S_ of winter wheat^5–9^. Conventional tillage in successive years has been found to harden and tighten the soil layers below 30 cm, leading to the formation of the bottom layer of plough, the enhancement of the soil permeability and porosity, and reduction of soil storage water capacity. This is unsuitable to the root growth and the water absorption and utilization, resulting in a great decline in the crop yields and WUE^10^. Deep ploughing can promote the crop growth and increase the grain yields through deepening the plough layer, broking the hard plough layer, and improving the soil layer structures at 30–40 cm, the soil porosity^11^, the soil water infiltration capacity and storage water capacity, and the soil storage water capacity and WUE^12^. Zhang and his colleagues have found that both irrigation periods and amounts significantly affect the wheat grain yields and WUE^13^. Even if the total irrigation amounts are same, different irrigation methods and irrigation water distributions during different growth stages also have significant effects on the wheat yields and WUE^14,15^. Water consumption and WUE of winter wheat are remarkably changed by other factors, such as wheat varieties^16,17^, tillage managements^18–20^, irrigation systems (irrigation amounts, periods and frequencies)^21–23^, and fertilizers^24,25^. Totally, these previous studies focused on the effects of single or double factors and their interactions on WUE of winter wheat. To our knowledge, however, the effects of comprehensive agronomic managements including sowing, water and fertilizer management, etc. on the grain yields and water use of winter wheat remain unclear. In the present study, we conducted a field trait, in which four combined cultivation managements including tillage, row spacing configuration, water and fertilizer inputs and planting densities, was set up to aim at their effects on the water consumption characteristics, WUE and grain yields of winter wheat. And we propose a hypothesis that combination of the above mentioned agronomic managements could effectively improve the grain yields and water use of winter wheat.

## Results

### Total water consumption and water composition of different sources of winter wheat

This experiment was conducted in two successive seasons of wheat growth in 2008.10.01–2009.06.10 and 2009.10.01–2010.06.10, in which appeared serious drought and low temperature, respectively (Table 1). The four cultivation managements had remarkable effects on the total water consumption amounts and the water source compositions, and the differences were significant among four cultivation managements (*P* < 0.05 or *P* < 0.01) (Table 2). In two wheat growth seasons (2008–2009 and 2009–2010), amounts of the total water consumption among the four cultivation managements showed similar patterns: T3 > T1 > T2 > T4. Compared with T1, T2, and T4, average amounts of the total water consumption in T3 during the two seasons increased by 4.8%, 11.2%, and 22.6%, respectively (Table 2). Compared with T1 and T4, however, this parameter in T2 significantly decreased by 5.8% and 18.4%, respectively. There were different profiles on amounts of the soil water consumption between T1 and T2, insignificant and significant differences in the drought year (2008–2009) and the low-temperature year (2009–2010), respectively. Compared with T3, amounts of the soil water consumption in T4 decreased significantly by average 38.6% in two seasons (Table 2).

**Table 1:** Meteorological parameters at two successive wheat growth seasons from October 2008 to June 2010 at our experimental region.

Meteorological factors	Year	Growth season								
		October	November	December	January	February	March	April	May	First 10 days in June
Average temperature (°C)	2008–2009	16.58(+1.35)	9.43(+1.36)	2.64(+0.68)	−0.17(−0.37)	5.36(+2.56)	9.23(+0.91)	15.86(+0.75)	20.73(−0.02)	25.75(−0.70)
	2009–2010	17.44(+2.14)	4.90(−3.17)	1.92(−0.14)	0.03(−0.17)	3.16(+0.43)	8.04(−0.25)	13.28(−1.83)	20.99(+0.26)	22.46(−2.59)
Precipitation (mm)	2008–2009	16.60(−23.20)	15.20(−5.50)	0.20(−7.80)	0.00(−7.80)	30.40(+19.70)	32.40(+10.40)	30.30(+3.00)	83.90(+37.20)	17.40(+2.10)
	2009–2010	13.10(−26.70)	39.40(+18.70)	1.40(−6.60)	0.10(−7.70)	8.80(−1.90)	9.50(−12.50)	47.40(+14.10)	25.90(−20.80)	22.60(+7.30)
Duration of sunshine (h)	2008–2009	168.70(−11.60)	161.50(+6.90)	173.60(+18.50)	152.40(+2.10)	73.90(−76.20)	172.40(−10.40)	213.80(+10.40)	208.90(−31.40)	85.90(+8.00)
	2009–2010	146.30(−34.00)	128.10(−32.50)	145.10(−10.00)	122.80(−33.50)	72.70(−77.40)	145.60(−37.20)	201.30(−8.10)	232.80(−7.50)	48.00(−29.90)

Note: Data in the brackets represent the difference in meteorological factors between the year and a normal year (1962–2006).

**Table 2:** Total water consumption and water composition in four managements.

Year	Managements	Amounts of total water consumption (mm)	Amounts of precipitation water (mm)	Amounts of irrigation water (mm)	Amounts of soil water consumption (mm)	Ratio to total water consumption (%)		
						Precipitation	Irrigation	Soil water
2008–2009	T1	507.5 bB	168.2	270.0	69.3 cC	33.1 cC	53.2 aA	13.6 dB
	T2	482.9 cC	168.2	240.0	74.7 cC	34.8 bB	49.7 bB	15.5 cB
	T3	536.1 aA	168.2	240.0	127.9 aA	31.4 dD	44.8 cC	23.8 aA
	T4	447.1 dD	168.2	180.0	98.9 bB	37.6 aA	40.3 dD	22.1 bA
2009–2010	T1	545.9 bB	226.4	270.0	49.5 bB	41.5 cC	49.5 aA	9.1 cBC
	T2	509.4 cC	226.4	240.0	43.0 cB	44.4 bB	47.1 bB	8.4 cC
	T3	567.0 aA	226.4	240.0	100.6 aA	39.9 dD	42.3 cC	17.7 aA
	T4	452.3 dD	226.4	180.0	45.9 bcB	50.1 aA	39.8 dD	10.1 bB

Note: The experimental data were evaluated using analysis of variance (ANOVA) and correlation analysis with SPSS 16.0, and multiple comparisons were conducted for significant effects using the least significant difference (LSD) test at α = 0.05 and α = 0.01.

Because water consumption sources in crops are composed of irrigation water, precipitation, and soil storage water, we compared their proportions in the total water consumption amounts in our experiment. Our data showed that the former two components accounted for higher proportions (76.2%~91.6%) of the total water consumption amounts in the wheat field, whereas that of soil storage water was merely 8.4%~23.8%. In both the dry year and the low-temperature year, the proportion of precipitation in T4 was 6.5%, 4.2% and 8.2% higher than T1, T2 and T3, respectively. Additionally, proportion of precipitation in T2 was 6.2% higher than T1, and T4 was 23.0% higher than T3 (Table 2). The proportion of irrigation water of the total water consumption in T1 was 2.9%, 7.8%, and 11.3% higher than those in T2, T3 and T4, respectively (Table 2). The highest proportion of soil storage water appeared in T3, 9.4%, 8.8% and 4.7% higher than T1, T2 and T4, respectively (Table 2).

### Water consumption characteristics in different growth stages of winter wheat

Different cultivation managements significantly changed the water consumption, daily water consumption and water consumption coefficient at different growth stages of wheat plants (Table 3). In two tested years, water consumption amounts was greatest at the middle (from jointing to anthesis, JTA) and late (from anthesis to maturity, ATM) growth stages of wheat plants under all managements, while least at the stage of wintering to resuming (WTR). After wintering, the daily water consumption and water consumption coefficient gradually increased in four managements with the proceeded growth stages (Table 3). Compared with the WTR stage, water consumption at the JTA and ATM stages increased by from 1.1- to 2.8-fold and 1.3- to 3.5-fold, respectively, and the water consumption coefficient increased by from 12.4% to 22.0% and 14.8% to 26.6%, respectively. Comparison among four managements indicated that the highest water consumption and daily water consumption at each growth stage occurred in T3 and T1 and differences among four managements increased gradually with the proceeded growth stages. Water consumption and daily water consumption at JTA stage in T1 management were higher than T2, T3, and T4 by 8.5%, 2.4%, and 17.1%, respectively (Table 3). These two parameters at ATM stage in T3 management were higher than T1, T2, and T4 by 7.8%, 12.4%, and 58.3%, respectively (Table 3). From wintering to anthesis (WTA) stages, the water consumption coefficient in T4 management was higher than that of T3, but significantly declined at the ATM stage by 6.9%. However, there was insignificant differences in the water consumption coefficient between T1 and T2 managements at all stages, except for JTA stages.

**Table 3:** Water consumption characteristics in different wheat growth stages.

Year	Managements	Emergence to wintering			Wintering to growth resuming			Growth resuming to jointing			Jointing to anthesis			Anthesis to maturity		
		WC (mm)	DWC (mm)	WCC (℅)	WC (mm)	DWC (mm)	WCC (℅)	WC (mm)	DWC (mm)	WCC (℅)	WC (mm)	DWC (mm)	WCC (℅)	WC (mm)	DWC (mm)	WCC (℅)
2008–2009	T1	102.4 bB	1.6 bB	20.2 aA	52.1 aA	0.8 aA	10.3 bB	73.0 cC	2.1 cC	14.4 bB	128.4 aA	4.0 aA	25.3 aA	151.5 aAB	4.0 aAB	29.8 aA
	T2	99.6 bB	1.5 bB	20.6 aA	49.8 aA	0.8 aA	10.3 bB	71.9 cC	2.1 cC	14.9 bB	117.0 bAB	3.7 bBC	24.2 abAB	144.6 bB	3.8 bB	29.9 aA
	T3	111.4 aA	1.7 aA	20.8 aA	51.4 aA	0.8 aA	9.6 cC	91.0 aA	2.6 aA	17.0 aA	125.0 abA	3.9 aAB	23.3 bB	157.3 aA	4.1 aA	29.4 aA
	T4	92.7 cC	1.4 cC	20.7 aA	51.5 aA	0.8 aA	11.5 aA	78.2 bB	2.2 bB	17.5 aA	107.1 cB	3.3 cC	24.0 abAB	117.6 cC	3.1 cC	26.3 bB
2009–2010	T1	86.9 abAB	1.3 aAB	15.9 bB	48.8 aA	0.8 aA	8.9 bB	74.7 bAB	2.8 bB	13.7 cC	162.0 aA	3.9 aA	29.7 abAB	173.4 bB	4.4 bB	31.8 bA
	T2	79.7 cC	1.2 bB	15.6 bB	39.5 cC	0.6 cC	7.8 cC	71.9 cC	2.7 cC	14.1 bcBC	151.2 bAB	3.6 bAB	29.7 abAB	167.1 bB	4.3 bB	32.8 abA
	T3	89.1 aA	1.3 aA	15.7 bB	43.1 bB	0.7 bB	7.6 cC	82.2 aA	3.0 aA	14.5 bB	158.7 abA	3.8 aA	28.0 bB	193.9 aA	5.0 aA	34.2 aA
	T4	85.4 bB	1.2 aAB	18.9 aA	42.4 bB	0.7 bB	9.4 aA	76.7 bB	2.8 bB	16.9 aA	141.7 cC	3.4 cB	31.3 aA	106.1 cC	2.7 cC	23.5 cB

Notes: WC, water consumption; DWC, daily water consumption; WCC, water consumption coefficient. The experimental data were evaluated using analysis of variance (ANOVA) and correlation analysis with SPSS 16.0, and multiple comparisons were conducted for significant effects using the least significant difference (LSD) test at α = 0.05 and α = 0.01.

**Figure 1:**
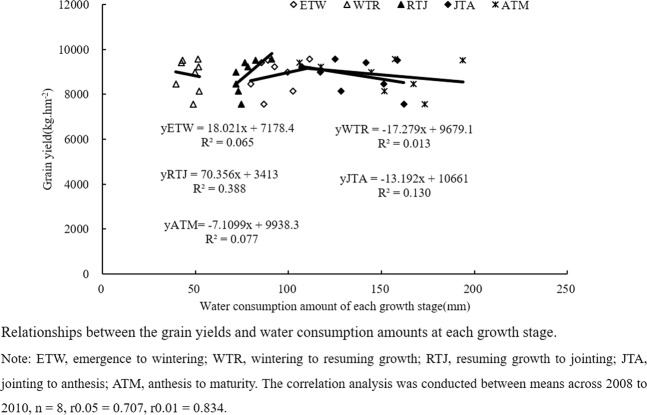
Relationships between the grain yields and water consumption amounts at each growth stage.

The relationship between the grain yields and water consumption amounts at each growth stage of winter wheat differed among four managements (Fig. 1). There was a positive correlation between the grain yields and water consumption amounts at ETW and RTJ stages, while negative correlation occurred at WTR, JTA and ATM stages with R^2^JTA > R^2^ATM > R^2^WTR (Fig. 1). This finding demonstrated that reducing water consumption after the jointing stage could be used as an efficient mean to improve wheat yields.

### Water consumption of the different soil layers

**Figure 2:**
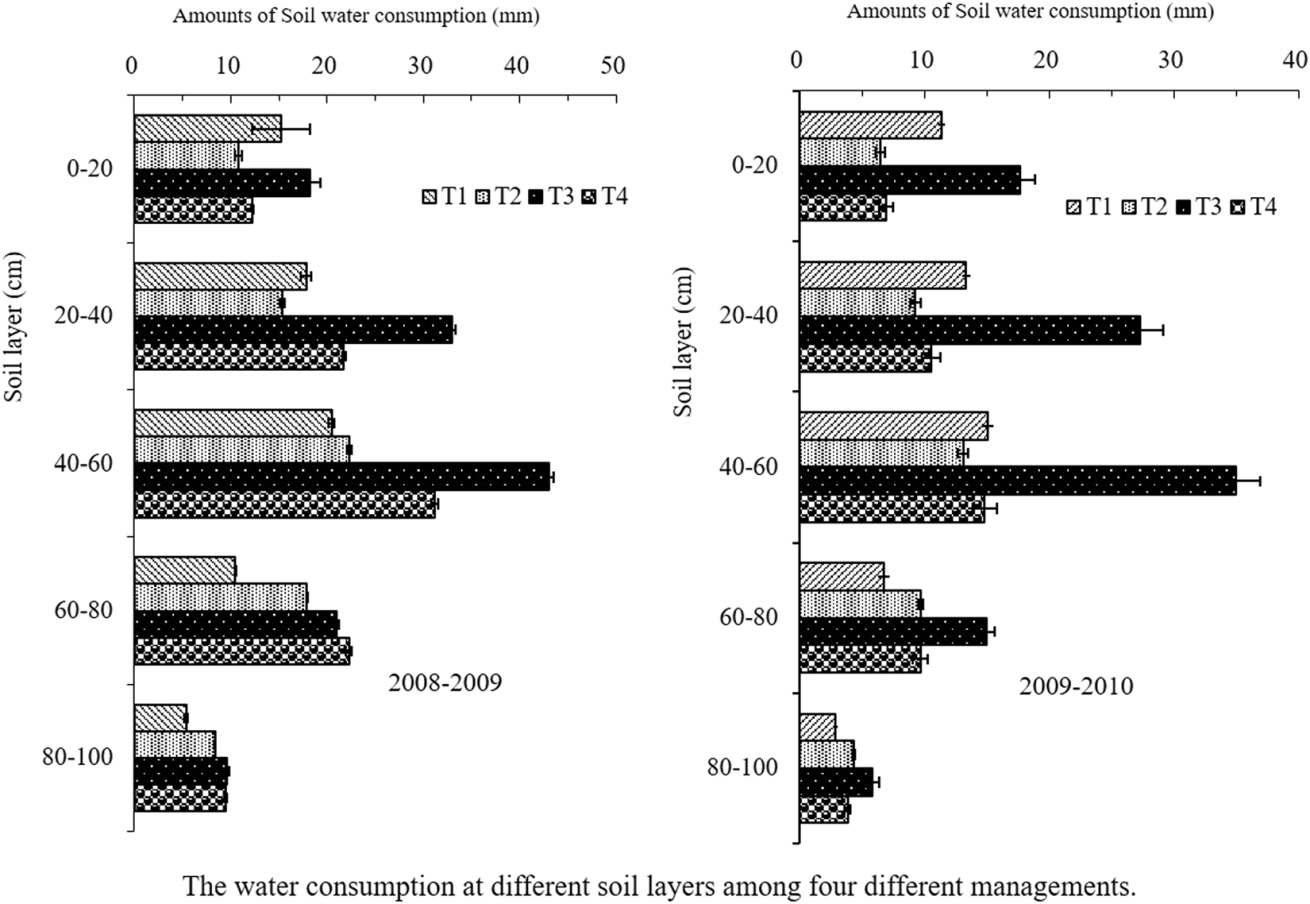
The water consumption at different soil layers among four different managements.

Among the four cultivation managements, the highest soil water consumption amounts appeared at the 40–60 cm soil layer and at this layer, the proportion was up to 29.5%-34.4% (Fig. 2). Soil water consumption amounts at 80–100 cm layer, however, was lower and its proportion was only from 5.6% to 11.2% (Fig. 2). Soil water consumption amounts at 0–20 cm and 20–40 cm layers in T1 and T3 were significantly higher than those in T2 and T4, respectively, whereas this parameter below 60 cm soil layer decreased significantly (Fig. 2). Water consumption amounts above 40 cm soil layer in T2 management was significantly lower than T1 management, and T4 was also lower than T3 management (Fig. 2). Average reduction rates of water consumption amounts in T2 management declined by 28.5% compared to that of T1, with T4 compared to T3 by 47.3% (Fig. 2). Compared to T1, soil water consumption amounts at 60–80 cm and 80–100 cm soil layers in T2 management increased by 57.1% and 54.3%, respectively (Fig. 2). This parameter at 80–100 cm soil layer in T3 and T4 managements exhibited differential profiles during the two growth seasons. In the dry year (2008–2009), soil water consumption amounts at 80–100 cm soil layer in T4 were higher than T3 by 6.1%. In the low-temperature year (2009–2010), however, the former were lower than the latter by 35.1%. These suggested that T1 and T3 managements absorbed more and less water from the upper and the lower soil layers, respectively, possibly from root hydrotropism. In T1 and T3 managements, irrigated water mainly was storaged at the upper soil layer, leading to appearance of roots mainly at this soil layer. The optimized managements (T2 and T4) with reduced irrigation amounts and optimized irrigation periods helped promote the roots to grow at middle and lower soil layers and enhance the absorption and utilization at these two soil layers (Fig. 2).

### Yield and water use efficiency

Different cultivation managements had differential regulatory effects on the grain yields and WUE (Table 4). In two successive tested years, the grain yields, WUE_Y_, WUE_P_, WUE_I_ and IE parameters in T1 management were least among all managements in two tested years, and decreased by ≥2.3%, 13.8%, 10.0%, 20.0% and 54.2% lower than other three treatments, respectively (Table 4). Compared with T1, WUE_Y_, WUE_S_, WUE_P_ , WUE_I_ and IE in T2 remarkably increased by 18.0%, 15.6%, 11.1%, 25.0%, and 118.04%, respectively (Table 4). Compared with T3, WUE_Y_, WUE_S_, WUE_I_ and IE in T4 also increased by 19.8%, 70.9%, 30.2% and 21.1%, respectively (Table 4). However, there was insignificant difference in WUE_P_ between T3 and T4 managements. Grain yields in T3 were highest, whereas its WUE_S_ markedly decreased by 37.0%, 44.7% and 36.9% in comparison to T1, T2 and T4, respectively (Table 4). These suggested that T3 management primarily increased the uptake and utilization of irrigation water, while significantly decreased the uptake and utilization of soil storage water. T2 and T4 with reduced irrigation amounts and optimized irrigation periods helped enhance WUE_S_ and WUE_I_ demonstrating that enhancing the uptake and utilization of irrigation water and soil storage water could be used as a primary mean to improve WUE.

**Table 4:** Yield and water use efficiency.

Year	Managements	GY (kg·hm^−2^)	WUE_Y_ kg·hm^−2^·mm^−1^	WUE_S_ kg·hm^−2^·mm^−1^	WUE_P_ kg·hm^−2^·mm^−1^	WUE_I_ kg·hm^−2^·mm^−1^	IE kg·hm^−2^·mm^−1^
2008 –2009	T1	8136.5 cC	16.0 dC	117.6 aA	48.4 cC	30.1 dD	3.3 cC
	T2	8997.4 bB	18.6 bB	120.6 aA	53.5 bB	37.5 cC	7.3 bB
	T3	9573.79 aA	17.9 cB	75.2 cC	56.9 aA	39.9 bB	9.7 aA
	T4	9229.79 bAB	20.6 aA	93.7 bB	54.9 aAB	51.3 aA	11.0 aA
2009–2010	T1	7566.18 cC	13.9 cC	153.0 bB	33.4 cC	28.0 dD	3.6 dD
	T2	8452.56 bB	16.6 bB	196.8 aA	37.3 bB	35.2 cC	7.7 cC
	T3	9519.90 aA	16.8 bB	94.8 cC	42.0 aA	39.7 bB	12.2 bB
	T4	9416.04 aA	20.8 aA	206.0 aA	41.6 aA	52.3 aA	15.7 aA

Notes: GY, grain yield; WUE_Y_, yield water use efficiency; WUE_S_, soil water use efficiency; WUE_P_, precipitation water use efficiency; WUE_I_, irrigation water use efficiency; IE, irrigation effectiveness. The experimental data were evaluated using analysis of variance (ANOVA) and correlation analysis with SPSS 16.0, and multiple comparisons were conducted for significant effects using the least significant difference (LSD) test at α = 0.05 and α = 0.01.

Grain yields were significantly and positively correlated to WUE_Y_, WUE_P_ , WUE_I_ and IE (*P* < 0.05), while negative correlation appeared at WUE_S_ in two tested years (*P* < 0.05) (Table 5). WUE_Y_ was also significantly and positively correlated with WUE_P_ , WUE_I_ and IE (*P* < 0.05), while the correlation between WUE_Y_ and WUE_S_ was insignificant in two tested years (Table 5). In addition, there was a remarkable and negative correlation between WUE_S_, and WUE_P_ , WUE_I_ as well as IE (*P* < 0.01), whereas there were significant and positive correlations among the WUE_P_ , WUE_I_ and IE (*P* < 0.01) (Table 5). These showed that improving WUE_P_ , WUE_I_ , IE and WUE_Y_ could simultaneously increase WUE_S_, while the excessive use of soil water would greatly decline wheat grain yields.

**Table 5:** Relationships between grain yields and water use efficiency.

Year	Parameters	GY	WUE_Y_	WUE_S_	WUE_P_	WUE_I_	IE
2008–2009	GY	1.000					
	WUE_Y_	0.645^**^	1.000				
	WUE_S_	−0.708^**^	−0.243	1.000			
	WUE_P_	1.000^**^	0.646^**^	−0.708^**^	1.000		
	WUE_I_	0.679^**^	0.946^**^	−0.494	0.680^**^	1.000	
	IE	0.918^**^	0.854^**^	−0.669^**^	0.918^**^	0.910^**^	1.000
2009–2010	GY	1.000					
	WUE_Y_	0.785^**^	1.000				
	WUE_S_	−0.154	0.452	1.000			
	WUE_P_	1.000^**^	0.783^**^	−0.156	1.000		
	WUE_I_	0.836^**^	0.987^**^	0.322	0.835^**^	1.000	
	IE	0.940^**^	0.926^**^	0.105	0.939^**^	0.962^**^	1.000

Notes: GY, grain yield; WUE_Y_, yield water use efficiency; WUE_S_, soil water use efficiency; WUE_P_, precipitation water use efficiency; WUE_I_, irrigation water use efficiency; IE, irrigation effectiveness. ns indicates non-significant. * and ** indicate significant differences at the p-levels of 0.05 and 0.01, respectively. n = 6. P (t0.05) = 2.571, P (t0.01) = 4.032.

## Discussion

With the increased amounts of irrigation water in wheat fields, the total water consumption amounts in all soil layers significantly increase, whereas they quickly decrease in lower soil layer^26–28^. Zheng and his colleagues have found that deep ploughing can increase the total water consumption amounts and increase the consumed amounts of soil storage water^29^. In this experiment, total water consumption amounts in T1 was significantly higher than both T2 and T4, whereas its proportion of soil storage water was significantly lower than other three managements. The proportions of soil storage water in T3 and T4 were also significantly higher than those of T1 and T2, possibly from deep ploughing (Table 2). These findings are similar to the results of previous studies^26–29^. However, the total water consumption amounts in T3 was significantly higher than T1 and T2, and the proportion of soil storage water in T3 was also higher than T4, probably because deep ploughing and high input of water and fertilizer promoted wheat plants in this management grow vigorously after the jointing stage and wheat plants consumed a substantial amounts of water due to transpiration. After we comprehensively evaluated the total water consumption amounts and the water source compositions, the optimized managements of T2 and T4 were characterized with the reduced available soil water at planting and the delayed fertilization and irrigation to the jointing in spring season, resulting in the significantly reduced total water consumption amounts, the increased consumption of precipitation and soil storage water, and the reduced demand for irrigation water (Table 2).

After comparing the amounts of total water consumption, daily water consumption and water consumption coefficient at different growth stages in two successive tested years, we found that amounts of total water consumption and daily water consumption were highest at JTA and ATM stages in all managements, while least at the WTR stage. After wintering stage, the daily water consumption and water consumption coefficient gradually increased in four managements (Table 3). Cui and his colleagues have found that, after anthesis, lower soil layer water was the primary source of supplied water for requirement of wheat plants^30^. Therefore, improving the utilization of lower soil layer water can ensure a sufficient water supply during the grain filling stage. In this experiment, the effects of water consumption amounts of the different soil layers revealed that the consumed amounts of lower soil layer water in T2 management were significantly higher than those in T1, while this parameter in T4 was higher than that in T3 in only 2008–2009. The stages, amounts, and methods of irrigation in T4 need to be optimized (Fig. 2).

Previous studies have shown that added irrigation at the jointing and filling stages could effectively delay flag leaf senescence, and increase the WUE, grain filling rates, and the grain yields^31,32^. However, water deficit during these two stages can lead to a serious decline in the grain yields^33^. Xu and his colleagues have found that reducing the irrigation frequencies before the jointing stage could promote the growth of wheat roots into lower soil layers and increase the absorption of lower soil layer water, resulting in the improved WUE^34^. In addition, irrigation at the jointing stages to the flowering stages of winter wheat has been found to increase the grain yields, harvest index and WUE^35^. These suggest that irrigation can obviously affect the water utilization of wheat directly during different growth stages. In this study, the grain yields of T2, T3, and T4 were significantly higher than those of T1, and WUE_Y_, WUE_P_, WUE_I_ and IE in T4 were also higher than those of other three managements (Table 4). WUE_Y_, WUE_P_, WUE_I_ and IE in T2 were higher compared to T1, similar to the results in some previous studies^31–35^. In this experiment, the grain yields of T3 were highest because of quality-high soil tillage and sowing, enough water and fertilizer supply, and excessive input. Under the above conditions, however, the water use efficiency parameters in T3 were lower than those of the T4 (Table 4). After evaluating on the high water utilization efficiency and grain yields, thus, T4 was considered to be an optimized management with effectively utilizing the precipitation. However, T4 consumed a lower number of total soil storage water amounts, in particular from lower soil layer, than T2 and T3. These suggest that the irrigation water amounts, irrigation methods and mechanism of increasing grain yields and WUE in T4 management need to be optimized and explored.

## Conclusions

Deep ploughing, appropriately reducing amounts of fertilizers and irrigation, nitrogen fertilizer application and adding irrigation at jointing stage in T2 or T4 managements were beneficial methods to improve the absorption and utilization of irrigation and soil storage water in winter wheat, and promote the improvement of grain yields and WUE. These comprehensive managements could help for sustainable development of winter wheat with economizing fertilizers, increasing grain yields, and efficiency-high water utilization.

## Materials and Methods

### Experimental set-up

#### Field experiments

We conducted a field experiment at two successive wheat growth seasons from October 2008 to June 2010 at Xiangyun Town, Wenxian County, Henan Province, China (112°99′E, 34°92′N). There is a warm temperate semi-humid continental monsoon climate in this region, in which soil is fluvo–aquic clay soil, and other properties of the 0–40 cm plough-layer soil have been indicated in our previous study^36^ shown in Table 1. Continuous wheat-maize rotation model was used in these two growth seasons, and all the straws were returned to the field after harvest. A semi-winter bread wheat variety Ping’an 8, characterized with high yield potential, excellent biotic and abiotic tolerance, was used in this study. This variety was developed Henan Ping ‘an Seed Industry Co., Ltd., and was released in 2011.

Several combined managements (e.g., tillage, row spacing configuration, water and fertilizer inputs and planting densities) were considered and four treatments were designed as follows: traditional management (T1), optimized management compared with T1 (T2), super high-yield management (T3), and optimized management compared with T3 (T4) (Table 6). For T1, rotary tillage (approximately 15 cm-depth) was performed, whereas rolling was not conducted before sowing. The planting densities of the wheat seeds were 187.5 kg·hm^−2^ with 20 cm-width equal row spacing and irrigation was applied after sowing. For T2, T3 and T4, mechanical deep ploughing (over 25 cm-depth) was adopted, soil was harrowed 2–3 times, and rolling was conducted after sowing. Equal row spacing (20 cm-width) was adopted in T2, while alternating wide- and narrow row spacing (15 cm × 23 cm width) was adopted in T3 and T4. The planting densities of the wheat seeds in T2 were 150 kg·hm^−2^, whereas 120 kg·hm^−2^ in both T3 and T4. Organic and microelement fertilizers, and phosphate and potassium fertilizers were applied before sowing (Table 6). As the base fertilizers, all nitrogen (N) fertilizers in T1 were applied before sowing one time, while in T2, T3 and T4, 50% of the N fertilizers were applied before sowing, and the remaining 50% were applied at the jointing stages in combined with irrigation. In each wheat growth season, a randomized complete block design and a factorial arrangement of treatments were used with four biological replicates with 50 m^2^ each. Other managements were the same as local standard wheat practices. In addition, control was established for the whole wheat growth stage without irrigation to calculate the IE, where in each control area was 20 m^2^, and other inputs were consistent with those of four managements. Sowing was conducted on October 15 for the two wheat growth seasons. Wheat grains were harvested on June 7, 2009, and June 11, 2010, respectively.

**Table 6:** Irrigation and fertilization management of wheat under different cultivation managements.

Managements	Amounts of fertilizer application (kg·hm^−2^)					Irrigation water stages and amounts (m^3^·hm^−2^)			
	N	P_2_O_5_	K_2_O	ZnSO_4_	Organic fertilizer	Soil moisture	Green rose	Jointing	Blossom filling
T1	225	75	60	0	0	900	900	0	900
T2	180	75	60	0	0	600	0	900	900
T3	300	150	150	15	3000	600	0	900	900
T4	240	90	90	15	3000	600	0	600	600

#### Growth conditions

There were some differences in climate conditions in two wheat growth seasons (Table 1). From the beginning of October to the end of January in 2008–2009, precipitation was only 32 mm, 44.76 mm less than the average precipitation in normal years. Especially from December 2008 to January 2009, there was merely 0.2 mm precipitation, 15.48 mm less than the average precipitation in normal years. Thus, there occurred a serious drought stress during the early growth stages in this wheat planting season. In 2009–2010, there occurred severe low temperature stress (0.87 lower than average values in previous successive 45 wheat growth seasons, from 1962–2006) in winter and spring seasons. This stress occurred early and lasted for a long stage (from the beginning of November to the end of April) in this season (Table 1). Especially in November this year, average temperature was only 4.90 °C, 3.22 °C lower than average values. Throughout the growth stage, the total accumulated temperature was 106.90 °C lower than average values. The duration of sunshine decreased by 268.0 h, primarily because of a 117.5 h decrease in February and March 2010.

### Measurements

#### Soil water

Soil samples were randomly drilled at three points in each plot before sowing and at the main wheat growth stages. Each 20 cm-depth soil was considered as one layer, and 1 m-depth soil was sampled for 3 times at each plot. Then the sampled soil layers were mixed in one plot and immediately placed in an aluminium box. For the determination of the soil water content, wet soil samples were first weighed and baked in a 110 °C oven for 10–12 h to constant weights and then weighed again. Equation (1) was used to calculate the soil quality water content.

1 $$\theta =\frac{W1-W2}{W2}\times 100$$

*θ*, *W1*, and *W2* represent the soil water content (%), the wet and dry soil weights (g), respectively.

#### Total water consumption and soil storage water

According to previous study^32^, total soil water consumption was determined with Equation (2):

2 $$\begin{array}{ll}\Delta S={10}{\rm{\Sigma }}{\gamma }_{i}{H}_{i}({\theta }_{il}\,-\,{\theta }_{i2}) & i({1},n)\end{array}$$

*Δ*S, *i*, *n*, *γ*_*i*_, *Hi*, *θ*_*il*_, and *θ*_i2_ represent the soil water storage consumption (mm), the number of solum, total soil layers, soil dry bulk density of layer *i* (g.cm^−3^), the thickness of the soil (cm), the soil water content at the beginning of layer *i*, and the soil moisture at the end of layer *i*, respectively. Values are expressed as percentage (%) of the dry soil weight.

Soil storage water was calculated based on the method of Liu and his colleagues^37^, and this parameter was determined using Equation (3):

3 $$E{T}_{1-2}=10\mathop{\sum }\limits_{i=1}^{n}{\gamma }_{i}{{\rm{{\rm H}}}}_{i}({\theta }_{i1}-{\theta }_{i2})+I+{\rm{{\rm P}}}+{\rm{{\rm K}}}\,{\rm{i}}(1,\,{\rm{n}})$$

*ET*_1–2_ is water consumption amounts at stage (mm), and *i*, n, γ_i_, H_i_, θ_i1_, *θ*_i2_ are the same as those in Equation (2). *I*, P, and *K* represent the total irrigation amounts (mm), precipitation (mm), and increment of groundwater (mm), respectively. When the depth of groundwater is over 2.5 m, the K value can be neglected. In this study. The depth is under 5 m, and the K can be regarded as 0.

#### WUE

WUE_Y_, WUE_I_, WUE_P_, WUE_S_, and IE were determined using Equations (4–8), respectively.

4 $$WU{E}_{Y}=Y/ET$$

5 $$WU{E}_{I}=Y/I$$

6 $$WU{E}_{P}=Y/P$$

7 $$WU{E}_{S}=Y/\Delta S$$

8 $$IE=({Y}_{i}-{Y}_{ni})/I$$

Units of WUE_Y_, WUE_I_, WUE_P_, and WUE_S_ are kg·hm^−2^·mm^−1^; *Y*, *P*, *ΔS*, *ET*, *I*, *Y*_*i*_, and *Y*_*ni*_ represent the yields (kg·hm^−2^), precipitation (mm), amounts of the soil water consumption (mm), amounts of total water consumption (mm), irrigation amounts, grain yields in irrigation management (kg·hm^−2^), and yields in non-irrigation management (kg·hm^−2^).

#### Grain yields and components

At maturity stage, wheat plants in 10 m^2^ area were harvested in each experimental plot, and the harvested samples were threshed and dried to calculate the grain yields (kg·hm^−1^).

### Statistical analysis

The experimental data were evaluated using analysis of variance (ANOVA) and correlation analysis with SPSS 16.0, and multiple comparisons were conducted for significance using the least significant difference (LSD) at 0.05 and 0.01 levels.
